# Safety of Deep Repetitive Transcranial Magnetic Stimulation (drTMS) against Medical Refractory Symptoms in Parkinson Syndromes: First German Real-World Data with a Specific H5 Coil

**DOI:** 10.3390/neurolint14040082

**Published:** 2022-12-12

**Authors:** Celine Cont, Annaliis Lehto, Nathalie Stute, Anastasia Galli, Christina Schulte, Veronika Deer, Michaela Wessler, Lars Wojtecki

**Affiliations:** 1 Department of Neurology and Neurorehabilitation, Hospital zum Heiligen Geist, Academic Teaching Hospital of the Heinrich-Heine-University Düsseldorf, 47906 Kempen, Germany; 2 Institute of Clinical Neuroscience and Medical Psychology, Medical Faculty, Heinrich-Heine-University Düsseldorf, 40225 Düsseldorf, Germany; 3 Translational Neurodegeneration Section “Albrecht Kossel”, Department of Neurology, University Medical Center Rostock, University of Rostock, 18147 Rostock, Germany; 4 Deutsches Zentrum für Neurodegenerative Erkrankungen (DZNE) Rostock/Greifswald, 18147 Rostock, Germany

**Keywords:** deep transcranial magnetic stimulation, Parkinson Syndrome, adverse events, adverse device effect, real-world data, patients

## Abstract

So far, deep repetitive transcranial magnetic stimulation (drTMS) has shown promising results as an add-on treatment for Parkinson’s disease (PD) but not for non-idiopathic Parkinson Syndromes (PS). We aimed to investigate the safety and feasibility of drTMS application in patients with different Parkinson Syndromes and medical refractory symptoms. Multifaceted real-world data (*n* = 21) were retrospectively analyzed regarding adverse effects as well as short-term effects of the drTMS treatment on patients’ self-rated symptom severity and motor, cognitive, and emotional functions. The drTMS treatment with H5 coil included a sequential 1 Hz primary motor cortex stimulation contralateral to the more-affected body side and a bilateral 10 Hz stimulation of the prefrontal cortex. Overall, drTMS could be safely administered to patients with different PSs and medical refractory symptoms, but large variation was apparent in the rate and severity ratings of the reported adverse event/adverse device effect. The treatment significantly decreased the subjective main symptom severity. This effect was more pronounced in older patients with PD. Furthermore, analysis showed an improvement in depression, but no effect could be established in terms of cognitive performance. drTMS can be safely administered to patients with PS and medical refractory symptoms and can decrease the subjective motor symptom severity and depression.

## 1. Introduction

Current treatment options of levodopa-refractory symptoms in the later stages of Parkinson’s disease (PD) and especially non-idiopathic Parkinson Syndromes (PS) remain suboptimal for several patients. In these patients, the initial beneficial effect of medication is difficult to maintain, and over the years patients often develop debilitating refractory symptoms such as freezing of gait [1,2]. In advanced PD, dopaminergics are a common therapeutic option, while levodopa optimization is critical [2]. Another pharmacological treatment includes the addition of an MAO-B inhibitor; a COMT inhibitor; a DA; or an extended-release levodopa formulation, safinamide, and DA- or Levodopa pumps. Moreover, invasive deep brain stimulation has a high level of evidence in advanced treatment options [2]. While novel non-dopaminergic disease-modifying pharmacotherapies and cellular therapies are under development [3], non-invasive neurostimulation approaches, such as repetitive transcranial magnetic stimulation (rTMS), have surfaced as an add-on treatment option to pharmacotherapy [4,5].

Deep TMS (drTMS) is a type of rTMS applied with an H-coil that generates less focal magnetic fields than the commonly used figure-of-eight coil [6]. One advantage of the H-coil compared to the figure-of-eight coil is the slower decay of magnetic fields, which therefore reach deeper and stimulate a larger proportion of the brain [6,7,8]. Furthermore, the H5 coil in particular, designed for PD (Brainsway Inc., Jerusalem, Israel), can be used to bilaterally target motor cortex regions and/or the prefrontal cortex [9,10,11]. This is in accordance with findings from rTMS studies, which suggest that bilateral stimulation has the highest efficacy on motor symptoms in PD [5]. As an add on therapy for PD, drTMS has been reported to improve motor functions, autonomic and depressive symptoms, and activities of daily living, e.g., [9,10,12]. However, the application of drTMS in the treatment of other Parkinson Syndromes (PS) or patients with medical refractory symptoms has not yet been investigated.

Adverse device effects (ADEs) associated with drTMS have been studied in the context of various indications. The most common potential ADEs of drTMS include headaches, discomfort at the stimulation site, and facial discomfort during stimulation [13]. Various studies assessing the tolerability and effectiveness of drTMS in PD [10,11,12] have reported the forementioned ADEs and added dizziness, nausea, and sleepiness to the list. All these effects are generally reported as transient and mild. A further risk of rTMS is to trigger a seizure, although this occurs rarely and often in combination with other risk factors such as preexisting epilepsy, changes to the medication dosage, or alcohol consumption [13]. Therefore, drTMS seems to offer a safe and tolerable add-on therapy for PD patients, which encourages further investigation of its utility in treating refractory symptoms in the broader group of PS patients. The stimulation protocol that is most effective for PD is still unclear. While some prefer stimulating with high frequency of the prefrontal cortex (PFC) and low frequency of the motor cortex [12,14], some stimulate with high frequency of both [9,10]. Additionally, an evidence-based guideline on the therapeutic use of rTMS suggests targeting the bilateral motor cortex with high frequency [5]. However, several studies reported an impaired intra-cortical inhabitation in PD patients, which highlights the importance of low-frequency stimulation of the primary motor cortex (for a summary, see the introduction in [12]). Moreover, high-frequency stimulation of the prefrontal cortex has been shown to increase striatal dopamine release, which causes an improvement of motor symptoms (for a summary, see the introduction in [12]). The stimulation protocol for this study used low-frequency stimulation of the motor cortex as well as high-frequency stimulation of the prefrontal cortex.

The current research entails a retrospective analysis of real-world data regarding drTMS application in a clinical context. Namely, existing multifaceted information gathered as part of standard clinical practice in the neurology department of the Hospital zum Heiligen Geist was analyzed according to specific hypotheses. The data came from a heterogeneous sample of consecutive patients with different neurodegenerative forms of PS and medical refractory symptoms, who differed from each other in terms of diagnosis, age, treatment goal, and levels of symptom severity. Taking place in the first center using the H5 coil in Europe, this investigation aimed to examine the safety and feasibility of drTMS treatment with this coil in PS. The hypotheses for this analysis included the following: (i) drTMS application is safe and not associated with severe adverse events or device effects; (ii) drTMS can be successfully applied to patients regardless of their diagnosis, age, impairment profile, and symptom severity; (iii) drTMS application improves motor and cognitive functions as well as patients’ subjective symptom severity.

## 2. Materials and Methods

### 2.1. Patients

Patient data (*n* = 21, 13 males, 8 females) from the Hospital zum Heiligen Geist in Kempen, Germany were analyzed. The exclusion criteria for receiving the drTMS treatment included diagnosed epilepsy, pregnancy, presence of an implanted pacemaker or other metal implants, and alcohol and/or drug use on the day before or on the day of the stimulation. The inclusion criteria for the stimulation treatment included (i) a diagnosis of a PS and (ii) refractory hypokinetic or tremor symptoms from levodopa medication or the need for reduction in levodopa dose due to side effects from the medication. The diagnostic criteria for PS were bradykinesia and at least one of the following features: rest tremor, muscular rigidity, or disturbances of posture and gait. The main treatment goal or symptom for each patient was defined before stimulation.

Before the start of the treatment, patients consented to receiving the stimulation treatment according to the CE-mark of the system in a real-world setting. Patient also consented to various data being recorded in an anonymous registry (Ethic Commission Number 2021026 Ärztekammer Nordrhein, 22 February 2021). The criteria for inclusion in the current analysis consisted of the patient’s assignment to drTMS treatment with H5 coil according to the manufacturer’s (Brainsway Inc., Jerusalem, Israel) treatment protocol for Parkinson’s. The majority of the patients had a diagnosed PD (*n* = 16); others had atypical neurodegenerative forms of PS (e.g., progressive supranuclear palsy, multiple system atrophy, and combined motor neuron disease) or mixed forms of PD with symptomatic PS. Although multiple patients received more than one drTMS treatment cycle, only the data regarding their first treatment cycle were included in the current analysis.

Some characteristics of the sample are summarized in Table 1. The estimated severity of motor impairments for the patients in this sample is indicated by the average score of the motor evaluation of the Unified Parkinson’s Disease Rating Scale (MDS-UPDRS III) when off dopaminergic medication (*n* = 15, M = 37.3, SD = 10.9) and on dopaminergic medication (*n* = 15, M = 25.9, SD = 16.6). Although some patients formally responded to levodopa in the MDS-UPDRS III, all patients had a main refractory symptom or side effect by medication according to the above-named inclusion criteria for stimulation. Due to the retrospective creation of the data registry, not all patient data are complete.

### 2.2. Materials

The neuropsychological tests and questionnaires, which were obtained as part of the standard assessment, are outlined below. Before the start of each stimulation session, patients evaluated the severity of their main symptom during the last 24 h on a numeric rating scale (NRS) ranging from 0 to 10, with higher numbers indicating higher intensity. Furthermore, they were asked to report and rate any side effects they had experienced since the last stimulation session on an identical NRS.

Movement Disorder Society (MDS)–sponsored Unified Parkinson’s Disease Rating Scale (UPRDS). This instrument is a widely used clinical rating scale for PD [15]. The third subscale, the motor examination (MDS-UPDRS III), is administered as part of the standard assessment to indicate patients’ motor abilities and levodopa response before drTMS. For some patients, the scores were available also from the day after completing the treatment.

Montreal Cognitive Assessment (MoCA). This widely used screening test assesses a variety of cognitive functions and has been rated as a recommended cognitive scale for PD [16]. MoCA is administered on the starting day of the drTMS treatment before the first stimulation and a parallel version of this test is administered immediately after the last stimulation.

Beck Depression Inventory-II (BDI-II). This questionnaire is a widely used measure in both research and clinical practice for assessing depression. BDI-II is administered in the same assessment with MoCA.

Beck Depression Inventory—Fast-Screen (BDI-FS). This brief self-report inventory comprising seven items is used to evaluate depression in patients whose behavioral and somatic symptoms attributable to medical problems may confound diagnosis. This instrument has been found to have good psychometric properties and its use in screening PD patients has been encouraged [17]. The BDI-FS replaced the BDI-II as the depression instrument halfway through the data collection.

### 2.3. Stimulation

**Figure 1:**
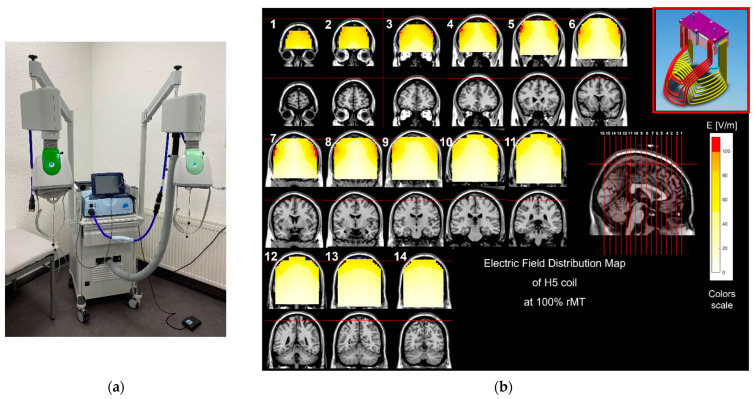
(**a**) Deep TMS system at the Department of Neurology in Kempen with Brainsway H5 Coil CE-marked for Parkinson’s (light green, right) and H2 Coil CE-marked for Alzheimer’s (dark green, left); Magstim Stimulator and Air-cooling. (**b**). Distribution of electric field induced by the H5 coil. The electric field distribution was measured in a model of the human head (15 × 13 × 18 cm) filled with physiologic saline solution. The colored field maps indicate the electric field absolute magnitude in each pixel, for 14 coronal slices, 1 cm apart, along with the appropriate MRI coronal images. The H5 coil was placed over the theoretical frontal cortex of the head model and the field in each pixel was measured using a ‘pick-up’ dipole probe attached to an oscilloscope. The red pixels indicate field magnitude above the threshold for neuronal activation, which was set to 100 V/m based on the average threshold for motor activation of the hand. The field maps are adjusted to obtain 100% of the threshold at a depth of 1.5 cm (Image provided by Brainsway).

After screening for contraindications for drTMS and completion of the neuropsychological assessment, the patients received between 5 and 11 treatments on consecutive workdays. The standard protocol of the manufacturer suggests 12 treatments over 4 weeks on an outpatient basis. Due to inpatient treatment, this protocol was adjusted and compressed to a shorter timeframe. Number of sessions was determined by the treating physician after considering individual clinical factors such as effect, side effects, and patients’ preferences. A dTMS H5 coil (Brainsway Inc., Jerusalem, Israel) was used with a MagStim stimulator (MagStim Company, Ltd., Whitland, UK, see Figure 1a,b).

The helmet with the H5 coil was positioned above the hemisphere contralateral to the symptom-dominant side. The hotspot of stimulation was determined by moving the helmet on the anterior–posterior and lateral–medial planes and monitoring the muscle activity of the appropriate index finger by using an EMG (resting motor threshold, RMT). When the hotspot with the highest muscle response was located, the position of the helmet was recorded according to the two measurement tapes fastened to the cap from anterior to posterior and from the left side to the right side and the position was used for the M1 stimulation. For the subsequent PFC stimulation, the helmet was centered and moved 6 cm anterior. The stimulation intensity was calculated according to the manufacturer’s guidelines (Brainsway Inc., Jerusalem, Israel), and the manufacturer’s stimulation protocol was followed. This consisted of a 1 Hz M1 stimulation centered on the more-affected hemisphere and a consequent bilateral 10 Hz PFC stimulation as also used by other studies [12,14]. The M1 stimulation was standard at 90% intensity from the resting motor threshold (RMT) intensity and consisted of 900 pulses applied at 1 s intervals. The PFC stimulation was standard at 100% (RMT) intensity and consisted of 40 trains of 20 pulses applied with 20 s intervals between the trains and amounting to 800 pulses. The calculated stimulation intensity was too uncomfortable for 15 out of 21 patients; so, the intensity was lowered until the patients could tolerate the treatment (see Table 2). Intensity values are stated below in Section 3.1.

### 2.4. Data Processing and Analysis

All the data were processed using Excel software and several hypotheses were addressed. Firstly, the data were screened for any treatment cancellations. Additionally, the presence, frequency, and severity of AE—respectively, ADEs—were assessed, and the rate of ADE was calculated per patient. Secondly, correlational analyses were carried out between the severity and rate of ADE and various personal and stimulation characteristics. Lastly, the short-term effects of drTMS on motor and cognitive functions were examined through one-tailed paired samples t-tests on the self-rated main symptom intensity and MoCA scores with alpha = 0.05 for significance.

## 3. Results

### 3.1. Safety of drTMS and AE/ADEs

The drTMS treatment was stopped in one out of twenty-one patients. The stimulation was stopped shortly after starting the first session due to patient’s discomfort. During the stimulation application, the patient’s face turned pale and he experienced nausea and stimulus-locked facial muscle contractions, prompting the cancellation of the treatment. No seizure was prompted. This reaction may have been associated with the levodopa test completed by the patient earlier that day. Thus, it is unclear whether it should be accounted as ADE or rather as AE. Besides this exception, other patients tolerated the stimulation treatment well.

A total of 199 stimulation sessions were carried out and an AE was recorded in 25 of them (12.56%). A total of eight different ADEs (thus possibly related to the stimulation) were reported by the patients. The most common ADE was headache, which was reported 15 times in total across the whole treatment by seven patients, followed by nausea and discomfort of the eye region or tearing of the eyes during stimulation, both reported in total four times by three patients. The application site discomfort was reported once by two patients and the rest of the ADEs (shoulder pain, increased rest tremor, tiredness, sleeplessness, intensified drifting to the left when walking) were mentioned once across the treatment by one patient each.

**Figure 2:**
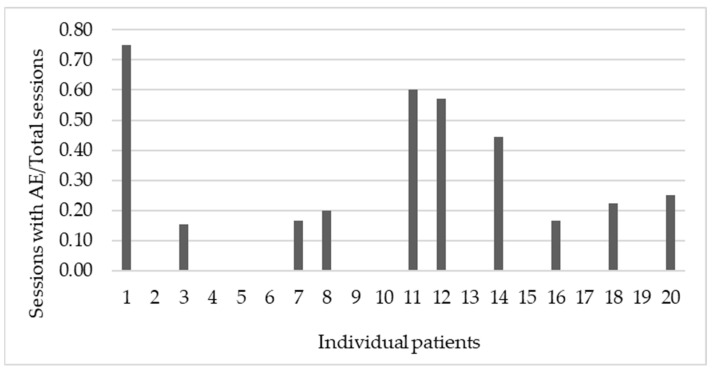
The y-axis represents the ratio between the number of sessions with AEs reported and the total number of stimulation sessions. The x-axis shows the individual patients displaying different levels of tolerability. The patients differed greatly in their reporting of AEs. Whereas some patients had a high ratio signifying frequent AE/ADEs, other patients did not report any AEs.

The intensity of AEs was rated subjectively on an NRS ranging from 0 to 10. A total of 10 from 20 patients (50%) reported at least 1 AE throughout the treatment and the severity of reported AEs varied greatly (M = 5.4, SD = 2.6). None of the AEs lasted beyond the day of stimulation. The ratio between the number of sessions where AEs were reported, and the total number of stimulation sessions was calculated per patient. The values of the ratio ranged from 0 to 0.75 (M = 0.2, SD = 0.2), as portrayed in Figure 2, displaying a differing level of tolerability between individuals.

Numerous correlational analyses were carried out to examine the relationships between the average AE severity and the rate of AEs on one hand and various personal and stimulation characteristics on the other hand. No relationship was found between the severity or rate of AEs and patients’ age, their self-reported main symptom severity before treatment, their cognitive level as indicated by their MoCA score before treatment, and their baseline main symptom severity according to the MDS-UPDRS III while on or off medication. In terms of stimulation parameters, the intensity of M1 and PFC stimulation were recorded (M = 47.6, SD = 8.5 and M = 46.3, SD = 7.8, respectively). The severity and rate of AEs were not associated with the M1-stimulation-intensity (r(36) = 0.06, *p* = 0.792; r(36) = 0.04, *p* = 0.885) nor the PFC-stimulation-intensity (r(38) = −0.16, *p* = 0.509; r(38) = 0.01, *p* = 0.981).

### 3.2. The Short-Term Effects on Main Symptom Severity and Cognitive Functions

**Figure 3:**
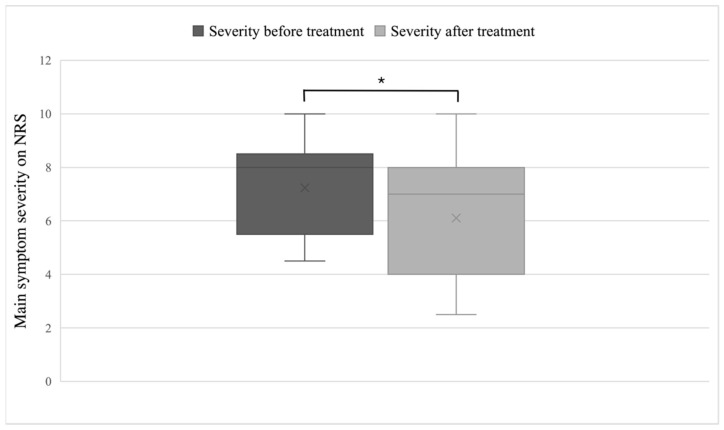
Mean of the patient group’s score of the main symptom severity measured on NRS before the treatment (dark grey) and after the treatment (light grey). The line represents the median of the group and the cross the mean scores. Despite the large amount of individual variation, the ratings on the NRS revealed a significant decrease in the severity of the main symptom from before to after the treatment (* *p* ≤ 0.05).

In general, a large individual variation was apparent in the change in main symptom severity, as evaluated subjectively on the NRS. The decrease in the severity of the patients’ main symptom, as evaluated subjectively on the NRS before (M = 7.2, SD = 1.8) and after the drTMS treatment (M = 6.1, SD = 2.2), was significant as evidenced by a one-tailed paired samples t-test (t(18) = −2.06, *p* = 0.027), shown in Figure 3. The number of sessions or the intensity of M1- or PFC-stimulation were not related to the change in main symptom severity in our sample (r(36) = 0.04, *p* = 0.866; r(34) = 0.03, *p* = 0.897; and r(36) = 0.15, *p* = 0.544, respectively).

The most common main symptom was hypokinetic gait, reported by 11 patients. The descriptive analysis of this subgroup revealed a large individual variation regarding change in the main symptom severity from before (M = 7.2, SD = 2.1) to after the drTMS treatment (M = 6.7, SD = 2.2).

The post-treatment on-medication MDS-UPDRS III scores were available for six PS patients and varied greatly (M = 34.5, SD = 19.2). Change from the baseline score could be calculated for four patients (Pre M = 31.8, SD = 9.4; Post M = 28.3, SD = 11). The descriptive analysis of depression symptoms as measured by BDI-II or BDI-FS before treatment (M = 10.6, SD = 7.0 and M = 2.3, SD = 2.3) and afterwards (M = 3.6, SD = 3.8 and M = 1.5, SD = 1.2) revealed a decrease or a maintenance in the reported symptoms in 10 from 11 PS patients. A one-tailed paired samples t-test revealed a significance difference before and after treatment in BDI-II (t(3) = −3.8, *p* = 0.015) but no significant difference in BDI-FS (t(4) = 1.2, *p* = 0.145). An overview is shown in Table 3.

**Figure 4:**
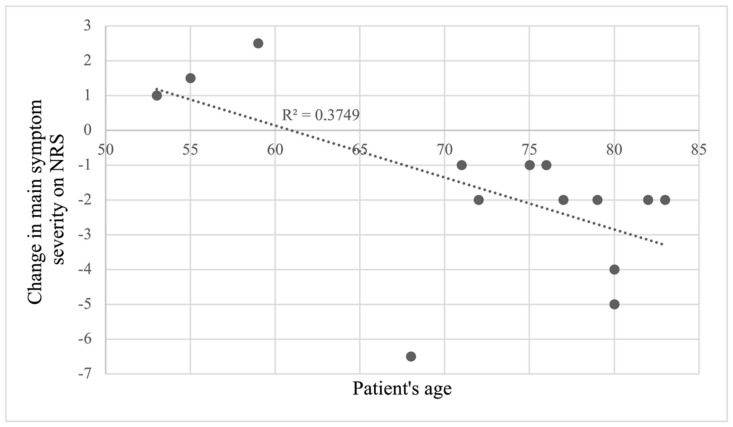
A correlation between the change in main symptom severity and patients’ age. The decrease in the main symptom severity was greater for older patients, whereas the younger patients did not seem to benefit from the treatment to the same degree and some even reported a worsening of symptom severity.

Some further analyses were conducted with available data from PD patients. No effect of drTMS on cognition as tested with MoCA before (M = 24.7, SD = 6.2) and after (M = 24.0, SD = 5.6) the treatment was found (t(13) = −0.62, *p* = 0.274). Moreover, changes in MoCA scores were not related to age, number of stimulation sessions, nor to changes in main symptom severity. Lastly, a correlation was found between the change in the main symptom severity and patients’ age (r(26) = −0.61, *p* = 0.020), depicted in Figure 4.

## 4. Discussion

The current research entailed the first retrospective real-world data analysis of the application of drTMS in a heterogeneous sample of patients with different forms of PS and medical refractory symptoms in a hospital setting. The study aimed to evaluate the safety and feasibility of this add-on therapy using the H5 coil for this patient population as well as to explore the short-term effects regarding subjective refractory symptom relief, motor and cognitive functions, and depression. We found that drTMS could be safely administered. AEs were recorded in 12.56% of sessions, with the most common AE reported as a headache, which has to be accounted as ADE. This shows that the profile of recorded AEs was congruent with previous literature, e.g., [11,12,13,18]. Both the frequency and the severity ratings of AEs varied largely from patient to patient and were not related to the examined personal and stimulation parameters. Therefore, the experience of AEs seems to be related to still uncovered personal factors. In summary, the number of AE is not low, but no SAE, long-lasting, or severe events occurred. Thus, drTMS is considered as safe.

The analysis of short-term treatment effects revealed a large individual variation regarding its benefits. The decrease in subjective main symptom severity was significant; however, the intensities of M1- or PFC-stimulation were not related to this improvement. Interestingly, PD patients’ age was correlated with the change in main symptom severity, indicating that older patients with PD reported larger decreases in symptom severity. This finding may reflect the previously reported results of drTMS being more beneficial for patients with higher MDS-UPDRS III scores and longer disease durations [11] since these variables could not be included in the current analysis. Moreover, a trend for improvement in motor symptoms could be demonstrated by an improvement in the mean score of the UPDRS-III ON scale. As the main symptom was gait hypokinesia, this symptom could have been underrepresented in the UPDRS-III and, thus, not sensitive enough to pick up treatment effect. A more walking-related score could have been more helpful. Still, the effect on subjective rating in walking abilities can be due to activating stimulation of the PFC in a sense of improvement in the executive control of walking.

Lastly, the analysis illustrated a significant decrease in depression scores for most PS patients using the BDI-II questionnaire but no effect of drTMS was found on cognition in PD patients. No significant improvement, but a trend of improvement, of the depressive symptom was found when using the BDI-FS scale, which could be explained by the shortness of the scale compared to the BDI-II questionnaire. This improvement of depressive symptoms could be due to the high-frequency stimulation of the prefrontal cortex, which has shown to achieve antidepressive effects [14].

This research offers numerous new insights. Firstly, it supports the safety and feasibility of utilizing drTMS treatment in a hospital setting for patients with different forms of PS and refractory symptoms. Secondly, it indicates a benefit of drTMS on refractory symptoms (especially hypokinesia/freezing of gait), particularly in older PD patients. Thirdly, it offers validation for the intense treatment schedule of stimulating on consecutive workdays, which is better suited for a hospital stay than previously reported schedules (e.g., [9,10,11]). However, the number of sessions needed are hard to conclude from our data. We suggest to start with five consecutive workdays when an inpatient protocol is chosen and then to perform an interim clinical examination. Another remaining question is the duration of the treatment effect. In our study, we found an immediate improvement in motor and depressive symptoms as well as in the subjective refractory symptom relief. However, the duration of that effect is still unclear. A study with a follow-up session 30 days after drTMS treatment showed a remaining significant improvement, suggesting that the treatment effect of the stimulation could last for numerous weeks [14]. Multiple further points of interest, however, remain to be explored. The most effective drTMS protocol, for instance, is still undetermined. Despite the successful use of low-frequency M1 stimulation in this and some other drTMS studies (e.g., [12]), various rTMS studies [5] and a recent drTMS study [9] have demonstrated a beneficial effect of high-frequency M1 stimulation. Moreover, more extensive investigation is needed to determine which types of PS besides PD benefit from drTMS the most.

The current findings must be considered while keeping in mind the retrospective nature of this research and the limitations associated with that. Firstly, due to the open label nature of the study and the lack of a control group comparison, the extent of a placebo effect on the patient cannot be determined. As the raters of the study were not blinded, a placebo effect on the raters also cannot be ruled out. Secondly, the small sample size and partly incomplete data limited the nature and strength of possible conclusions. Thirdly, the analyzed sample of patients was heterogenous in some respects while similar in other aspects such as high cognitive performance and low depression symptoms, which may have led to a ceiling effect. The reliance on self-reported measures and the restricted availability of an objective measure for motor functions must be taken into consideration. Future studies should investigate the number of sessions needed for an effect. This study showed that the subjective self-reported symptom severity improved, yet neuropsychological tests failed to assess this improvement. Future studies should reevaluate the assessments and add a more sensitive screening tool.

## 5. Conclusions

This retrospective analysis found the drTMS treatment to be generally well-tolerated by patients with different forms of PS and medical refractory symptoms. The treatment led to a decrease in self-rated symptom severity, especially in older PD patients, and in depressive symptoms.

## Figures and Tables

**Table 1:** This table summarizes various baseline characteristics for all consecutive patients with Parkinson Syndromes that received the stimulation treatment.

Patient	Age	Diagnosis	Sex	Main Symptom	MDS-UPDRS III off ^1^	MDS-UPDRS III on ^2^
1	79	PD-AR ^3^	Male	Rigidity		25
2	82	aPS ^4^	Male	Hypokinetic gait	25	
3	41	PD-AR	Male	Rigidity	11	1
4	59	PD	Male	Hypokinetic gait, Freezing	29	9
5	83	PD-AR	Female	Hypokinetic gait	38	18
6	68	PD-E ^5^	Male	Tremor	41	18
7	53	PD	Male	Hypokinetic gait	48	8
8	71	PD-D ^6^	Male	Tremor		68
9	72	aPS: PSP ^7^	Female	Hypokinetic gait	50	40
10	76	PD & sPS ^8^	Male	Hypokinetic gait		
11	80	PD-AR	Female	Finger/Hand Hypokinesia	39	
12	55	PD-E	Male	Tremor	31	19
13	80	aPS: CMND ^9^	Female	Hypokinetic gait		
14	72	PD & sPS	Male	Hypokinetic gait, Postural instability	40	20
15	77	aPS: probable MSA ^10^	Female	Upper extremity Hypokinesia	51	43
16	60	PD-AR	Female	Speech	48	38
17	82	PD-AR	Male	Hypokinetic gait		
18	77	PD-AR	Male	Cognition		
19	75	PD-AR	Male	Hypokinetic gait	29	27
20	76	aPS: possible PSP	Female	Finger/Hand Hypokinesia	44	29
21	80	PD-AR	Female	Hypokinetic gait	36	26

^1^ MDS-UPDRS III off = the score of the motor evaluation of the Unified Parkinson’s Disease Rating Scale when off medication; ^2^ MDS-UPDRS III on = the score of the motor evaluation of the Unified Parkinson’s Disease Rating Scale when on medication [15]; ^3^ PD-AR = Parkinson’s Disease with predominant symptoms of akinesia and rigidity; ^4^ aPS = atypical Parkinson’s syndrome; ^5^ PD-E = Parkinson’s Disease subtype of the equivalent type; ^6^ PD-D Parkinson’s Disease with Dementia; ^7^ PSP = Progressive Supranuclear Palsy; ^8^ sPS = symptomatic Parkinson’s syndrome; ^9^ CMND = Combined Motor Neuron Disease; ^10^ MSA = multiple system atrophy.

**Table 2:** This table shows the number of sessions as well as the intensity parameters (percent of stimulator output) of M1 and PFC stimulation. Please note that the stimulation intensity was calculated according to the manufacturer’s guidelines based on resting motor threshold but was adjusted if it was too uncomfortable (15 out of 21 patients).

Patient	Number of Sessions	Intensity M1	Intensity PFC
1	5	40	45
2	7	35	45
3	1	45	-
4	6	45	50
5	5	54	40
6	5	32	35
7	10	45	40
8	7	45	35
9	6	58	60
10	6	59	65
11	11	49	55
12	11	50	50
13	8	54	40
14	9	45	40
15	10	41	45
16	11	59	50
17	7	45	45
18	10	36	40
19	10	59	50
20	8	-	45
21	9	54	50

**Table 3:** This table shows the mean scores, standard deviations, and significance level (* *p* ≤ 0.05) of the assessments that were used. A significant effect was shown in the depressive score using the BDI-II questionnaire and in the subjective main symptom using the NRS. A trend for improvement can be seen in the UPDRS ON and BDI-FS.

Scale	Pre Mean (SD)	Post Mean (SD)	*p*
MDS-UPDRS III (ON)	31.8 (9.4)	28.3 (11)	0.115
Main Symptom Severity NRS	7.2 (1.8)	6.1 (2.2)	0.027 *
MoCA	24.7 (6.2)	24 (5.6)	0.274
BDI-II	10.6 (7)	3.6 (3.8)	0.015 *
BDI-FS	2.3 (2.3)	1.5 (1.2)	0.145

## Data Availability

The datasets used and/or analyzed during the current study are available from the corresponding author on reasonable request. The data are not publicly available due to privacy and ethical restrictions.

## Abbreviations

PD	Parkinson’s disease
PS	Parkinson Syndromes
AE	Adverse event
ADE	Adverse device effect
drTMS	deep repetitive transcranial magnetic stimulation
rTMS	repetitive transcranial magnetic stimulation
PFC	Prefrontal cortex
PD-AR	Parkinson’s Disease with predominant symptoms of akinesia and rigidity
aPS	Atypical Parkinson’s syndrome
PD-E	Parkinson’s Disease subtype of the equivalent type
PD-D	Parkinson’s Disease with Dementia
PSP	Progressive Supranuclear Palsy
sPS	symptomatic Parkinson’s syndrome
CMND	Combined Motor Neuron Disease
MSA	multiple system atrophy
MDS-UPDRS	Movement Disorder Society sponsored Unified Parkinson’s Disease Rating Scale
NRS	Numeric rating scale
MoCA	Montreal Cognitive Assessment
BDI-II	Beck Depression Inventory-II
BDI-FS	Beck Depression Inventory—Fast-Screen
